# Supplementary material for: Characterisation of the long-term physical and mental health consequences of SARS-CoV-2 infection: A systematic review and meta-analysis protocol
Source: PLoS One. 2022 Apr 5;17(4):e0266232. doi: 10.1371/journal.pone.0266232 (PMC8982872; doi:10.1371/journal.pone.0266232)
Supplement: S1 Appendix — (DOCX) [file pone.0266232.s001.docx]

**Appendix 1. Search Strategy**

| Database | Search Date | Search Terms | Filters | Records retrieved |
| --- | --- | --- | --- | --- |
| PubMed | 28-07-2021 | ((((("long covid" OR "chronic covid" OR "post covid syndrome" OR covid OR covid-19 OR "long haul" OR "post acute covid")) AND ((cough OR pain OR "shortness of breath" OR dyspn* OR palpitations OR heart failure OR "heart attack" OR "transient ischemia" OR "myocardial infarction" OR arrythmia OR stroke OR "cardiovascular accident" OR DVT OR "thromb*" OR PE OR "pulmonary embo*"))) AND ((fatigue OR weakness OR "joint pain" OR arthralgia OR "loss of smell" OR anosmia OR ageusia OR "loss of taste" OR seizure OR "visual problems" OR diplopia OR "peripheral neuropathy" OR "loss of sensation" OR paresthesia OR tinnitus OR diarrhea OR "loss of appetite" OR "muscle pain" OR myalgia ))) AND ((constipation OR nausea OR vomiting OR " abdominal pain" OR insomnia OR depression OR anxiety OR "decreased cognitive function"))) AND ((constipation OR nausea OR vomiting OR " abdominal pain" OR insomnia OR depression OR anxiety OR "decreased cognitive function")) Filters: in the last 1 year | 1 year | 502 |
| Web of Science | 28/07/2021 | (((TS=("long covid" OR "chronic covid" OR "post covid syndrome" OR covid OR covid-19 OR "long haul" OR "post acute covid")) AND TS=(cough OR pain OR "shortness of breath" OR dyspn* OR palpitations OR heart failure OR "heart attack" OR "transient ischemia" OR "myocardial infarction" OR arrythmia OR stroke OR "cardiovascular accident" OR DVT OR "thromb*" OR PE OR "pulmonary embo*")) AND TS=(fatigue OR weakness OR "joint pain" OR arthralgia OR "loss of smell" OR anosmia OR agnosia OR "loss of taste" OR seizure OR "visual problems" OR diplopia OR "peripheral neuropathy" OR "loss of sensation" OR paresthesia OR tinnitus OR diarrhea OR "loss of appetite" OR "muscle pain" OR myalgia )) AND TS=(constipation OR nausea OR vomiting OR " abdominal pain" OR insomnia OR depression OR anxiety OR "decreased cognitive function" AND constipation OR nausea OR vomiting OR " abdominal pain" OR insomnia OR depression OR anxiety OR "decreased cognitive function") | None | 782 |
| EBSCOhost | 28/07/2021 | "((((("long covid" OR "chronic covid" OR "post covid syndrome" OR covid OR covid-19 OR "long haul" OR "post acute covid")) AND ((cough OR pain OR "shortness of breath" OR dyspn* OR palpitations OR heart failure OR "heart attack" OR "transient ischemia" OR "myocardial infarction" OR arrythmia OR stroke OR "cardiovascular accident" OR DVT OR "thromb*" OR PE OR "pulmonary embo*"))) AND ((fatigue OR weakness OR "joint pain" OR arthralgia OR "loss of smell" OR anosmia OR ageusia OR "loss of taste" OR seizure OR "visual problems" OR diplopia OR "peripheral neuropathy" OR "loss of sensation" OR paresthesia OR tinnitus OR diarrhea OR "loss of appetite" OR "muscle pain" OR myalgia ))) AND ((constipation OR nausea OR vomiting OR " abdominal pain" OR insomnia OR depression OR anxiety OR "decreased cognitive function"))) AND ((constipation OR nausea OR vomiting OR " abdominal pain" OR insomnia OR depression OR anxiety OR "decreased cognitive function")) Apply equivalent subjects on 2021-07-28 04:49 PM" | None | 1500 |
| Science Direct | 29/07/2021 | "long covid" OR chronic covid AND consequences | 1 year | 1160 |
| Scopus | 29/07/2021 | TITLE-ABS-KEY ( ( ( ( ( ( "long covid" OR "chronic covid" OR "post covid syndrome" OR covid OR covid-19 OR "long haul" OR "post acute covid" ) ) AND ( ( cough OR pain OR "shortness of breath" OR dyspn* OR palpitations OR heart AND failure OR "heart attack" OR "transient ischemia" OR "myocardial infarction" OR arrythmia OR stroke OR "cardiovascular accident" OR dvt OR "thromb*" OR pe OR "pulmonary embo*" ) ) ) AND ( ( fatigue OR weakness OR "joint pain" OR arthralgia OR "loss of smell" OR anosmia OR ageusia OR "loss of taste" OR seizure OR "visual problems" OR diplopia OR "peripheral neuropathy" OR "loss of sensation" OR paresthesia OR tinnitus OR diarrhea OR "loss of appetite" OR "muscle pain" OR myalgia ) ) ) AND ( ( constipation OR nausea OR vomiting OR " abdominal pain" OR insomnia OR depression OR anxiety OR "decreased cognitive function" ) ) ) AND ( ( constipation OR nausea OR vomiting OR " abdominal pain" OR insomnia OR depression OR anxiety OR "decreased cognitive function" ) ) ) | None | 717 |
| Google Scholar | 29/07/2021 | long covid articles consequences OR impacts OR symptoms "long covid" OR "chronic covid" | None | 59 |
| EMBASE |  | To be searched | None |  |
| Psych INFO |  | To be searched | None |  |
