# Supplementary material for: Characterisation of the long-term physical and mental health consequences of SARS-CoV-2 infection: A systematic review and meta-analysis protocol
Source: PLoS One. 2022 Apr 5;17(4):e0266232. doi: 10.1371/journal.pone.0266232 (PMC8982872; doi:10.1371/journal.pone.0266232)
Supplement: Attachment — Submitted filename: R-PONE-D-21-25002.pdf [file pone.0266232.s003.pdf]

PLOS ONE

Characterisation of the long-term physical and mental health consequences of SARS-CoV-2 infection: a systematic review and meta-analysis protocol

--Manuscript Draft--

**Manuscript Number:**	PONE-D-21-25002
**Article Type:**	Study Protocol
**Full Title:**	Characterisation of the long-term physical and mental health consequences of SARS-CoV-2 infection: a systematic review and meta-analysis protocol
**Short Title:**	Characterisation of the long-term physical and mental health consequences of SARS-CoV-2 infection:
**Corresponding Author:**	Paul Kuodi, MPH<br>Bar-Ilan University Faculty of<br>Medicine Safed, Northern District<br>ISRAEL
**Keywords:**	Long Covid; Epidemiology; mental; psychosocial; social; impact; health
**Abstract:**	Background<br><br>As of July 2021, there has been more than 185 million documented cases of the novel coronavirus (SARS-CoV-2) infections and more than 4 million deaths globally. Despite more than 90%) of documented cases being classified as "recovered" from SARS- CoV-2 infection, a proportion of patients report a wide variety of persisting symptoms after the initial onset or acute phase of the infection, often referred to as "Long Covid". As data on the symptomatology of post-acute SARS-CoV-2 infection gradually becomes available, there is an urgent need to organise and synthesise the data in order to define what constitutes Long Covid and assist with its management in clinical and community settings.<br><br>Methods<br><br>This protocol follows the Preferred Reporting Items for Systematic Review and Meta-analysis Protocols (PRISMA-P) guidelines. A comprehensive literature search strategy will be developed in accordance with the Cochrane highly sensitive search guidelines. The following electronic databases will be searched for studies to include in the systematic review and meta-analysis: MEDLINE (via PubMed), Scopus, Google Scholar, Web of Science (Web of Knowledge), Science direct, and EBSCOhost. Dual screening will be applied at every screening stage. Two reviewers will independently screen titles, abstracts and full text of potentially eligible studies following the predefined inclusion and exclusion criteria in order to select studies to include in the review. As heterogeneity is anticipated between the included studies, data will be pooled in a meta-analysis using a random effects model. A clustering analytic approach will be applied to identify symptoms groupings and assign the symptoms into clusters. R statistical software will be used for the meta-analysis. Highly heterogeneous data will be synthesised narratively. The studies will be assessed, for quality using quality assessment tools appropriate for each study design. Two reviewers will independently undertake the quality of studies assessments.<br><br>Dissemination plans<br><br>Findings of the systematic review will be disseminated through a peer-reviewed publication and presentation of findings at conferences, workshops and government and private sector stakeholder engagement meetings.
**Order of Authors:**	Paul Kuodi, MPH
	Yanay Gorelik
	Michael Edelstein

Comment [A1]: remove)

Comment [A2]: reported

Powered by Editorial Manager® and ProduXion Manager® from Aries Systems Corporation

Additional Information:	
**Question**	**Response**

Powered by Editorial Manager® and ProduXion Manager® from Aries Systems Corporation

**Financial Disclosure**<br><br>Enter a financial disclosure statement that describes the sources of funding for the work included in this submission. Review the submission guidelines for detailed requirements. View published research articles from *PLOS ONE* for specific examples.<br><br>This statement is required for submission and **will appear in the published article** if the submission is accepted. Please make sure it is accurate.<br><br>**Unfunded studies**<br>Enter: *The author(s) received no specific funding for this work.*<br><br>**Funded studies**<br>Enter a statement with the following details:<br>• Initials of the authors who received each award<br>• Grant numbers awarded to each author<br>• The full name of each funder<br>• URL of each funder website<br>• Have or will the sponsors or funders play any role in the study design, data collection (when applicable) and analysis, decision to publish, or preparation of the manuscript?<br>• **NO** - Include this sentence at the end of your statement: *The funders had and will not have a role in study design, data collection and analysis, decision to publish, or preparation of the manuscript.*<br>• **YES** - Specify the role(s) played.<br><br>* typeset	3000

Powered by Editorial Manager® and ProduXion Manager® from Aries Systems Corporation

**Competing Interests**	No competing interests
Use the instructions below to enter a competing interest statement for this submission. On behalf of all authors, disclose any competing interests that could be perceived to bias this work—acknowledging all financial support and any other relevant financial or non-financial competing interests.<br><br>This statement is required for submission	

Powered by Editorial Manager® and ProduXion Manager® from Aries Systems Corporation

and **will appear in the published article** if the submission is accepted. Please make sure it is accurate and that any funding sources listed in your Funding Information later in the submission form are also declared in your Financial Disclosure statement.<br><br>View published research articles from *PLOS ONE* for specific examples.<br><br>**NO authors have competing interests**<br><br>Enter: *The authors have declared that no competing interests exist.*<br><br>**Authors with competing interests**<br><br>Enter competing interest details beginning with this statement:<br><br>*I have read the journal's policy and the authors of this manuscript have the following competing interests: [insert competing interests here]*<br><br>* typeset	
**Ethics Statement**<br><br>Enter an ethics statement for this submission. This statement is required if the study involved:<br><br>• Human participants<br>• Human specimens or tissue<br>• Vertebrate animals or cephalopods<br>• Vertebrate embryos or tissues<br>• Field research<br><br>Write "N/A" if the submission does not require an ethics statement.<br><br>General guidance is provided below. Consult the submission guidelines for detailed instructions. **Make sure that all information entered here is included in the Methods section of the manuscript.**	Ethical approval not relevant

Format for specific study types	
**Human Subject Research (involving human participants and/or tissue)**<br>• Give the name of the institutional review board or ethics committee that approved the study<br>• Include the approval number and/or a statement indicating approval of this research<br>• Indicate the form of consent obtained (written/oral) or the reason that consent was not obtained (e.g. the data were analyzed anonymously)<br><br>**Animal Research (involving vertebrate animals, embryos or tissues)**<br>• Provide the name of the Institutional Animal Care and Use Committee (IACUC) or other relevant ethics board that reviewed the study protocol, and indicate whether they approved this research or granted a formal waiver of ethical approval<br>• Include an approval number if one was obtained<br>• If the study involved *non-human primates*, add *additional details* about animal welfare and steps taken to ameliorate suffering<br>• If anesthesia, euthanasia, or any kind of animal sacrifice is part of the study, include briefly which substances and/or methods were applied<br><br>**Field Research**<br><br>Include the following details if this study involves the collection of plant, animal, or other materials from a natural setting:<br>• Field permit number<br>• Name of the institution or relevant body that granted permission	

Powered by Editorial Manager® and ProduXion Manager® from Aries Systems Corporation

**Data Availability**<br><br>Authors are required to make the data underlying their research findings fully available, without restriction.<br><br>If the manuscript reports pilot or preliminary results, the data underlying those results must be made available at the time of publication. A Data Availability	N/A - the protocol does not report results

Powered by Editorial Manager® and ProduXion Manager® from Aries Systems Corporation

Statement describing where the data can be found is required at submission. Your answers to this question constitute the Data Availability Statement and will be published in the article, if accepted. When legal or ethical requirements prohibit public data sharing, authors must indicate how researchers may obtain access to the data. See the PLOS Data Policy and FAQ for detailed information.<br><br>**Important:** Stating 'data available on request from the author' is not sufficient. If your data are only available upon request, select 'No' for the first question and explain your situation in the text box.<br><br>For protocols without pilot or preliminary data, authors are strongly encouraged to state how they plan to share research data from their study when it is completed or published.<br><br>Do the authors confirm that EITHER the protocol does not report results OR all data underlying the preliminary results will be made fully available without restriction?	
**Describe where the data may be found in full sentences. If you are copying our sample text, replace any instances of XXX with the appropriate details.**<br><br>• If **no pilot data are reported** in the manuscript, please enter the following: *No datasets were generated or analysed during the current study. All relevant data from this study will be made available upon study completion.*<br>• If the manuscript reports pilot data **held in a public repository**, include URLs, accession numbers or DOIs. If this information will only be available after acceptance, indicate this by ticking the box below. For example: *All XXX files are available from the XXX database*	No results as yet for this protocol

(accession number(s) XXX, XXX.).<br>• If the manuscript reports pilot data contained **within the manuscript and/or Supporting Information files**, enter the following:<br>*All relevant data are within the manuscript and its Supporting Information files.*<br>• If neither of these applies but you are able to provide **details of access elsewhere**, with or without limitations, please do so. For example:<br><br>*Data cannot be shared publicly because of [XXX]. Data are available from the XXX Institutional Data Access / Ethics Committee (contact via XXX) for researchers who meet the criteria for access to confidential data.*<br><br>*The data underlying the results presented in the study are available from (include the name of the third party and contact information or URL).*<br>• This text is appropriate if the data are owned by a third party and authors do not have permission to share the data.<br><br>For study protocols with data management or sharing plans, authors are encouraged to briefly describe in their Data Availability Statement how data they generate will be made accessible when the study is completed. For example:<br>• Deidentified research data will be made publicly available when the study is completed and published.<br><br>* typeset	
Additional data availability information:	

Powered by Editorial Manager® and ProduXion Manager® from Aries Systems Corporation

Manuscript Click here to access/download;Manuscript;Manuscript.doc

Characterisation of the long-term physical and mental health consequences of SARS-CoV-2 infection: a systematic review and meta-analysis protocol

Paul Kuodi[1, *], Yanay Gorelik[¶1], Michael Edelstein[¶1]

Authors' affiliations:

[1]Azrieli Faculty of Medicine, Bar-Ilan University, Safed, Israel

Correspondence to:

Paul Kuodi: kuodipa@biu.ac.il
The Azrieli Faculty of Medicine
Bar-Ilan University
8 Henrietta Szold St, Safed
Israel.

Abstract

Background: As of July 2021, there has been more than 185 million documented cases of the novel coronavirus (SARS-CoV-2) infections and more than 4 million deaths globally. Despite more than 90%) of documented cases being classified as "recovered" from SARS-CoV-2 infection, a proportion of patients report a wide variety of persisting symptoms after the initial onset or acute phase of the infection, often referred to as "Long Covid". As data on the symptomatology of post-acute SARS-CoV-2 infection gradually becomes available, there is an urgent need to organise and synthesise the data in order to define what constitutes Long Covid and assist with its management in clinical and community settings.

Methods: This protocol follows the Preferred Reporting Items for Systematic Review and Meta-analysis Protocols (PRISMA-P) guidelines. A comprehensive literature search strategy will be developed in accordance with the Cochrane highly sensitive search guidelines. The following electronic databases will be searched for studies to include in the systematic review and meta-analysis: MEDLINE (via PubMed), Scopus, Google Scholar, Web of Science (Web of Knowledge), Science direct, and EBSCOhost. Dual screening will be applied at every screening stage. Two reviewers will independently screen titles, abstracts and full text of potentially eligible studies following the predefined inclusion and exclusion criteria in order to select studies to include in the review. As heterogeneity is anticipated between the included studies, data will be pooled in a meta-analysis using a random effects model. A clustering analytic approach will be applied to identify symptoms groupings and assign the symptoms into clusters. R statistical software will be used for the meta-analysis. Highly heterogenous data will be synthesised narratively. The studies will be assessed, for quality using quality assessment tools appropriate for each study design. Two reviewers will independently undertake the quality of studies assessments.

Disseminationplans: Findings of the systematic review will be disseminated through a peer-reviewed publication and presentation of findings at conferences, workshops and government and private sector stakeholder engagement meetings.

Keywords: Long Covid, Epidemiology, mental, psychosocial, social, impact, health

PROSPERO registration number: CRD4202126589

Introduction

By July 2021, there were more than 185 million documented cases of the novel coronavirus (SARS-CoV-2) infections and more than 4 million deaths globally (1). The clinical course of SARS-CoV-2 infection ranges from asymptomatic to a fatal disease (2). Despite more than 90% of the documented cases being reported as "recovered" from SARS-CoV-2 infections (1), a proportion of patients continue to report persistent mild to severe symptoms after the initial onset or acute phase of the infection (2). Owing to its novelty, many aspects of the SARS-CoV-2 infection are still not well understood. Specifically, the nature, extent and pathogenesis of its post acute phase sequelae are yet to be well understood (3).

There is currently no full consensus among the scientific community about the pathway leading from SARS-CoV-2 infection to long term health consequences, a phenomenon commonly referred to as Long Covid or Chronic Covid syndrome (4). As yet, there is no agreement on the definition of post acute COVID-19 (2). An early attempt defined post acute COVID-19 as the clinical manifestation of symptoms that extend beyond 3 weeks following the initial symptoms of COVID-19 and Chronic Covid Syndrome (Long Covid) as clinical symptoms after initial onset of symptoms that extend beyond 12 weeks (5). The current definition of Long Covid has fundamental limitations which curtails its application in clinical settings. Firstly, the definition of Long Covid is broad and not tagged to specific symptoms that can help care providers come to a diagnosis of the condition. A wide range of symptoms are reported in literature with little attempt to identify which symptoms genuinely constitute a post-acute consequence of SARS-CoV-2 infection (6). Secondly, some studies reported Long Covid symptoms on patients who were never tested for SARS-CoV-2 infection in the first place (7). Thirdly, asymptomatic patients (which includes up to 50% of COVID-19 cases) have

largely been ignored in the reporting as research is currently focussed on hospitalised symptomatic cases.

The symptoms most commonly reported are fatigue, dyspnoea, joint and chest pain (8). In addition, organ specific symptoms primarily of the brain, heart and the lungs have been reported (9,10). Apart from the physical health consequences of SARS-CoV-2 infections, a host of psychosocial consequences of SARS-CoV-2 infection have also been reported (2). The long list of symptomatology of Long Covid, the course of the disease including timing of symptoms onset, as currently documented is not conducive to building a consensus on a specific case definition or towards its clinical diagnosis and management.

The proposed systematic review and meta-analysis will attempt to measure qualitatively and quantitatively, using available data, the consequences of SARS-CoV-2 infection on the physical, mental and social health domains.

As data on the symptomatology of post-acute SARS-CoV-2 infection gradually becomes available, there is an urgent need to organise and synthesise the data in order to define what constitutes and what does not constitute Long Covid and assist with its management in clinical and community settings. The review seeks to answer the research question: "*What are the long-term physical and mental health impacts of SARS-COV-2 infection on patients?".*

Methods

Study design

This protocol follows the Preferred Reporting Items for Systematic Review and Meta-analysis Protocols (PRISMA-P) guidelines (11). The systematic review topic has been

registered on the International Prospective Register of Systematic Reviews (PROSPERO) platform: Number: CRD4202126589 (12).

Search strategy

A search strategy will be developed in accordance with the Cochrane highly sensitive search guidelines (13). The search strategy will be piloted on PubMed database before conducting the literature search in different databases.

The search strategy will be developed using search terms and free-text words. Boolean operators 'AND' and 'OR' as follows; ("long covid" OR "chronic covid" OR "post covid syndrome" OR covid OR covid-19 OR "long haul" OR "post acute covid") AND (cough OR pain OR "shortness of breath" OR dyspn* OR palpitations OR heart failure OR "heart attack" OR "transient ischemia" OR "myocardial infarction" OR arrythmia OR stroke OR "cardiovascular accident" OR DVT OR "thromb*" OR PE OR "pulmonary embo*" OR fatigue OR weakness OR "joint pain" OR arthralgia OR "loss of smell" OR anosmia OR ageusia OR "loss of taste" OR seizure OR "visual problems" OR diplopia OR "peripheral neuropathy" OR "loss of sensation" OR paraesthesia OR tinnitus OR diarrhoea OR "loss of appetite" OR "muscle pain" OR myalgia OR constipation OR nausea OR vomiting OR " abdominal pain" OR insomnia OR depression OR anxiety OR "decreased cognitive function"). The search terms will be modified to include controlled descriptors (such as MeSH terms) and their synonyms. See **Appendix 2** for the provisional search strategy.

Comment [A3]: OR abdominal pain OR abdominal discomfort OR abdominal cramp OR
OR insomnia OR sleeplessness OR poor sleep, OR impaired sleep, OR lack of sle[illegible]
OR Decreased cognitive function OR delirium, OR acute generalized cerebral impairment, OR acute organic brain syndrome OR impaired consciousness, OR Seizure OR convulsion OR epilepsy OR Impaired vision, OR blind*

Information sources

The following electronic databases will be searched for studies to include in the systematic review and meta-analysis: MEDLINE (via PubMed), Scopus, Web of Science (Web of Knowledge), Science direct, Google Scholar, and EBSCOhost,

Comment [A4]: Ovid, Cochrane Library, search from gray literature, assessment of expert opinion

A hand-search of reference lists of included studies will be conducted. Organisations and consortiums with ongoing studies on Long Covid will be consulted to identify potential studies for inclusion in the review.

Study selection

The review question will *guide the* inclusion and exclusion criteria for studies to include in the systematic review and meta-analysis. To be eligible for inclusion the systematic review and meta-analysis, studies will have to be original research articles of peer-reviewed observational studies; cohort studies, cross-sectional studies, and case-control studies. Other study designs including case reports, case series, RCTs and reviews of all kinds will be excluded. To be included in the study, original research articles will have to report on participants who tested for SARS-CoV-2 through serological tests or molecular tests irrespective of inclusion or exclusion of control groups. Furthermore, studies will be included only if they report self-reported or verified symptoms of SARS-COV-2 infection after symptoms of the initial SARS-COV-2 infection have subsided, or new symptoms not SARS-COV-2 infection related or ongoing SARS-COV-2 infection symptoms after the acute phase. No time restriction will be applied on the time of onset of the symptoms provided they are symptoms appearing on recovered patients. Studies reporting on the mental health consequences of SARS-COV-2 infection will also be included.

Finally, we will restrict the literature search to only studies published in English language.

Screening and data extraction

Dual screening will be applied at every screening stage. Two reviewers will independently screen titles, abstracts and full text of potentially eligible studies following the predefined inclusion and exclusion criteria in order to select studies to include in the review. The two reviewers will in addition independently extract data from included studies, and grade the quality of evidence. At every stage of screening and quality of evidence grading, a third reviewer will be consulted if disagreements arise between the first and the second reviewers.

Data will be extracted using a data extraction tool designed specifically for this study and developed with considerations from the instructions provided by the Cochrane Collaboration (14). In order to identify the data items to be extracted, the proposed tool considers four elements for data extraction: (1) details of the study (article title; journal title; authors; country of the study; language; publication year; host institution of the study); (2) methodological characteristics (study design; study objective or research question or hypothesis; sample characteristics (e.g. sample size; sex; age, ethnicity; groups and controls; follow-up duration; validation of measures; statistical analyses); (3) main findings, and (4) conclusions.

Study outcomes

Two categories of outcomes of SARS-CoV-2 infection will be considered in this systematic review and meta-analysis 1) Physical Health Outcomes of SARS-CoV-2 infections (Respiratory outcomes- Cough, SOB, dyspnoea), (Cardiovascular outcomes-palpitations,

Heart attacks, arrythmias stroke, DVT, transient ischemia, pulmonary emboli, myocardial infarction, easy bleeding), (Musculoskeletal outcomes- fatigue, muscle weakness, joint pain, myalgia, arthralgia), (Neurological outcomes- syncope, Dizziness, seizures, tremors, diplopia, insomnia, ageusia, anosmia, tinnitus, loss of balance, pins and needles, loss of sensation, pain and discomfort, cognitive function deterioration), (Gastrointestinal outcomes- Abdominal pain, loss of appetite, constipation, diarrhoea, nausea and vomiting). 2) Mental Health Outcomes of SARS-CoV-2 infections- anxiety, depression, loss of self-care ability.

Study quality assessment

The studies will be assessed for quality using quality assessment tools appropriate for each study design. Two reviewers will independently undertake the quality of studies assessments and risk of bias scoring. Any discrepancy in the quality assessed by the reviewers will be resolved through discussion before arriving at a consensus. Publication bias will be assessed using funnel plots if at least 10 studies are included in the meta-analysis (15). and the findings reported as appropriate.

Descriptive analysis and meta-analysis

As it is anticipated that included studies will be heterogeneous given the diversity in the population of included participants, data will be pooled in a meta-analysis using a random effects model. R and STATA statistical software will be used for all data analysis. Highly heterogeneous data will be synthesised narratively. Heterogeneity will be assessed in the meta-analysis using Higgins & Thompson's I^2 Statistic and Predictive Intervals (PIs) with significance defined at the 10% α-level and quantified with the Higgins' I^2 statistic (15). I^2 statistics of between 25% and less than 50% will be considered low heterogeneity, I^2

statistics of between 50 % and less than 75% will be considered moderate heterogeneity and I^2 statistics of greater than 75% will be considered substantial heterogeneity.

Where data will be sufficient, subgroup analyses will be conducted by study design, participants characteristics, and by the clusters of outcomes of interest. If data will allow, unsupervised k-means clustering approach, will be applied to identify symptoms groupings and assign the symptoms into clusters. Sensitivity analyses will be carried out to examine the effect of risk of bias on the pooled estimates. Studies with high risk of bias scores will be excluded from the meta-analysis. In the event that heterogeneity will be very high, the data will be synthesised narratively.

The Grades of Recommendation Assessment, Development and Evaluation (GRADE) approach will be used to assess the certainty of the evidence (16). Meta-analysis will be performed using R-statistical software version 4.1.0 and STATA V.16 software (17).

Patient and public involvement

Patients will not be involved in the conduct of this systematic review and meta-analysis

Ethics and dissemination

The systematic review and meta-analysis will utilise publicly available literature and data. Hence, it will not require ethical approval.

Discussion

In many countries, the race to contain the SARS-COV-2 pandemic through mass vaccination of the population is still underway. Emerging evidence shows that SARS-COV-2 infection has long term health consequences in many domains of health. Recovery from SARS-COV-2 infection takes a protracted path in a group of SARS-COV-2 infected patients, a condition

that is now widely recognised as Long Covid. Consequently, a new public health problem is now emerging in the wake of SARS-COV-2 pandemic.

The characterisation of Long Covid, its frequency, severity, signs and symptoms and health outcomes are still under investigation. Yet evidence-based data is urgently needed to inform the health planning for the new Long Covid public health problem. This systematic review attempts to collate available data on this important subject in order to inform global Long Covid health policy directions.

Strengths and limitations of this study

- ☐ The systematic review and meta-analysis will be conducted and reported according to the guidelines of Preferred Reporting Items for Systematic Reviews and Meta-Analyses (PRISMA).
- ☐ A comprehensive literature search strategy has been developed. In addition, research organisations and consortiums with ongoing studies on long Covid will be consulted to identify potential studies for inclusion in the review.
- ☐ Literature search will be restricted to studies published in English language.
- ☐ The strength and quality of evidence from the review will be dependant on availability and quality of data on the review topic

Abbreviations

COVID-19- Coronavirus Disease 2019

SARS-COV-2- Severe Acute Respiratory Syndrome Coronavirus 2

GRADE- Grades of Recommendation Assessment, Development and Evaluation

SOB- Shortness of Breath

DVT- Deep Vein Thrombosis

MeSH- Medical Subject Headings

PROSPERO- Prospective Register of Systematic Reviews

PRISMA-P- Preferred Reporting Items for Systematic Review and Meta-analysis Protocols

Authors' contributions: ME conceived the study idea and provided conceptual guidance for the design of the protocol. PK designed the search strategy and wrote the first draft of the manuscript. ME and YG provided critical insights on the manuscript. All authors have approved and contributed to the final version of the manuscript.

Patient consent for publication: Not required.

Funding: No specific funding was received.

Competing interests: None declared.

Provenance and peer review: Not commissioned; externally peer reviewed.

Author information:

Paul Kuodi, Email: kuodipa@biu.ac.il , Azrieli Faculty of Medicine, Bar-Ilan University, Safed, Israel

Yanay Gorelik Email: yanay.gorelik@biu.ac.il , Azrieli Faculty of Medicine, Bar-Ilan University, Safed, Israel

Michael Edelstei, Email: michael.edelstein@biu.ac.il , Azrieli Faculty of Medicine, Bar-Ilan University, Safed, Israel

References

1. WHO Coronavirus (COVID-19) Dashboard | WHO Coronavirus (COVID-19) Dashboard With Vaccination Data [Internet]. [cited 2021 Jul 11]. Available from: https://covid19.who.int/

2. Del Rio C, Collins LF, Malani P. Long-term Health Consequences of COVID-19. JAMA - J Am Med Assoc. 2020 Nov 3;324(17):1723–4. doi:10.1001/jama.2020.19719

3. Mannan A, Mannan Baig A. Chronic COVID syndrome: Need for an appropriate medical terminology for long-COVID and COVID long-haulers. J Med Virol [Internet]. 2021 [cited 2021 Jul 12];93:2555–6. Available from: https://doi.org/10.1002/jmv.26624

4. Perego E, Callard F, Stras L, Melville-Jóhannesson B, Pope R, Alwan NA. Why the Patient-Made Term "Long Covid" is needed. Wellcome Open Res 2020 5224 [Internet]. 2020 Sep 24 [cited 2021 Jul 12];5:224. Available from: https://wellcomeopenresearch.org/articles/5-224

5. Greenhalgh T, Knight M, Court CA', Buxton M, Husain L. Management of post-acute covid-19 in primary care. Available from: http://dx.doi.org/10.1136/bmj.m3026

6. López-León S, Wegman-Ostrosky T, Perelman C, Sepulveda R, Rebolledo PA, Cuapio A, et al. More than 50 Long-Term Effects of COVID-19: A Systematic Review and Meta-Analysis. SSRN Electron J [Internet]. 2021 Feb 6 [cited 2021 Jul 25]; Available from: https://papers.ssrn.com/abstract=3769978

7. Tenforde MW. Symptom Duration and Risk Factors for Delayed Return to Usual Health Among Outpatients with COVID-19 in a Multistate Health Care Systems Network — United States, March–June 2020. MMWR Morb Mortal Wkly Rep [Internet]. 2020 Jul 31 [cited 2021 Jul 12];69(30):993–8. Available from: https://www.cdc.gov/mmwr/volumes/69/wr/mm6930e1.htm

8. Carfì A, Bernabei R, Landi F. Persistent symptoms in patients after acute COVID-19. JAMA - J Am Med Assoc. 2020 Aug 11;324(6):603–5. doi:10.1001/jama.2020.12603

9. Puntmann VO, Carerj ML, Wieters I, Fahim M, Arendt C, Hoffmann J, et al. Outcomes of Cardiovascular Magnetic Resonance Imaging in Patients Recently Recovered From Coronavirus Disease 2019 (COVID-19). JAMA Cardiol [Internet]. 2020 Nov 1 [cited 2021 Jul 12];5(11):1265–73. Available from: https://jamanetwork.com/journals/jamacardiology/fullarticle/2768916 doi:10.1001/jamacardio.2020.3557

10. Zhao Y miao, Shang Y min, Song W bin, Li Q quan, Xie H, Xu Q fu, et al. Follow-up study of the pulmonary function and related physiological characteristics of COVID-19 survivors three months after recovery. EClinicalMedicine. 2020 Aug 1;25. doi.org/10.1016/j.eclinm.2020.100463

11. Shamseer L, Moher D, Clarke M, Ghersi D, Liberati A, Petticrew M, et al. Preferred reporting items for systematic review and meta-analysis protocols (PRISMA-P) 2015: elaboration and explanation. BMJ [Internet]. 2015 Jan 2 [cited 2021 Jul 13];349. Available from: https://www.bmj.com/content/349/bmj.g7647

12. Booth A, Clarke M, Dooley G, Ghersi D, Moher D, Petticrew M, et al. The nuts and bolts of PROSPERO: an international prospective register of systematic reviews. Syst Rev 2012 11 [Internet]. 2012 Feb 9 [cited 2021 Jul 13];1(1):1–9. Available from: https://systematicreviewsjournal.biomedcentral.com/articles/10.1186/2046-4053-1-2

13. Lefebvre C, Manheimer E, Glanville J. Searching for studies. Cochrane Handbook for Systematic Reviews of Interventions. Cochrane B Ser. 2008;95–150.

14. O'Connor D, Green S, Higgins JPT. Defining the review question and developing criteria for including studies. Cochrane Handbook for Systematic Reviews of Interventions. Cochrane B Ser. 2008;81–94.

15. Higgins JPT, Altman DG, Sterne JAC. In: Higgins JPT, Green S (editors). Cochrane Handbook for Systematic Reviews of Interventions Version 5.1. 0 [updated March 2011]. The Cochrane Collaboration, 2011. Available from www. cochrane‐ handbook. org. 2011.

16. Goldet G, Howick J. Understanding GRADE: an introduction. J Evidence‐ Based Med. 2013;6(1):50–4.

17. StataCorp L. Stata statistical software: Release 15 (2017). Coll Station TX StataCorp LP. 2019.

326

328

327

Appendix 1. Search Strategy

Database	Search Date	Search Terms	Filters	Records retrieved
Scopus	29/07/2021	TITLE-ABS-KEY (((((("long covid" OR "chronic covid" OR "post covid syndrome" OR covid OR covid-19 OR "long haul" OR "post acute covid")) AND ((cough OR pain OR "shortness of breath" OR dyspn* OR palpitations OR heart AND failure OR "heart attack" OR "transient ischemia" OR "myocardial infarction" OR arrythmia OR stroke OR "cardiovascular accident" OR dvt OR "thromb*" OR pe OR "pulmonary embo*"))) AND ((fatigue OR weakness OR "joint pain" OR arthralgia OR "loss of smell" OR anosmia OR ageusia OR "loss of taste" OR seizure OR "visual problems" OR diplopia OR "peripheral neuropathy" OR "loss of sensation" OR paresthesia OR tinnitus OR diarrhea OR "loss of appetite" OR "muscle pain" OR myalgia)) AND ((constipation OR nausea OR vomiting OR "abdominal pain" OR insomnia OR depression OR anxiety OR "decreased cognitive function")) AND ((constipation OR nausea OR vomiting OR " abdominal pain" OR insomnia OR depression OR anxiety OR "decreased cognitive function")))	None	717
Google Scholar	29/07/2021	"long covid" articles consequences OR impacts OR symptoms "long covid" OR "chronic covid"	None	59
PubMed	28-07-2021	((((("long covid" OR "chronic covid" OR "post covid syndrome" OR covid OR covid-19 OR "long haul" OR "post acute covid")) AND ((cough OR pain OR "shortness of breath" OR dyspn* OR palpitations OR heart failure OR "heart attack" OR "transient ischemia" OR "myocardial infarction" OR arrythmia OR stroke OR "cardiovascular accident" OR DVT OR "thromb*" OR PE OR "pulmonary embo*")) AND ((fatigue OR weakness OR "joint pain" OR arthralgia OR "loss of smell" OR anosmia OR ageusia OR "loss of taste" OR seizure OR "visual problems" OR diplopia OR "peripheral neuropathy" OR "loss of sensation" OR paresthesia OR tinnitus OR diarrhea OR "loss of appetite" OR "muscle pain" OR myalgia))) AND ((constipation OR nausea OR vomiting OR " abdominal pain" OR insomnia OR depression OR anxiety OR "decreased cognitive function"))) AND ((constipation OR nausea OR vomiting OR " abdominal pain" OR insomnia OR depression OR anxiety OR "decreased cognitive function")) Filters: in the last 1 year	1 year	502
Web of Science	28/07/2021	(((TS=("long covid" OR "chronic covid" OR "post covid syndrome" OR covid OR covid-19 OR "long haul" OR "post acute covid")) AND TS=(cough OR pain OR "shortness of breath" OR dyspn* OR palpitations OR heart failure OR "heart attack" OR "transient ischemia" OR "myocardial infarction" OR arrythmia OR stroke OR "cardiovascular accident" OR DVT OR "thromb*" OR PE OR "pulmonary embo*")) AND TS=(fatigue OR weakness OR "joint pain" OR arthralgia OR "loss of smell" OR anosmia OR agnosia OR "loss of taste" OR seizure OR "visual problems" OR diplopia OR "peripheral neuropathy" OR "loss of sensation" OR paresthesia OR tinnitus OR diarrhea OR "loss of appetite" OR "muscle pain" OR myalgia)) AND TS=(constipation OR nausea OR vomiting OR " abdominal pain" OR insomnia OR depression OR anxiety OR "decreased cognitive function" AND constipation OR nausea OR vomiting OR " abdominal pain" OR insomnia OR depression OR anxiety OR "decreased cognitive function")	None	782
EBSCOhost	28/07/2021	"((((("long covid" OR "chronic covid" OR "post covid syndrome" OR covid OR covid-19 OR "long haul" OR "post acute covid")) AND ((cough OR pain OR "shortness of breath" OR dyspn* OR palpitations OR heart failure OR "heart attack" OR "transient ischemia" OR "myocardial infarction" OR arrythmia OR stroke OR "cardiovascular accident" OR DVT OR "thromb*" OR PE OR "pulmonary embo*"))) AND ((fatigue OR weakness OR "joint pain" OR arthralgia OR "loss of smell" OR anosmia OR ageusia OR "loss of taste" OR seizure OR "visual problems" OR diplopia OR "peripheral neuropathy" OR "loss of sensation" OR paresthesia OR tinnitus OR diarrhea OR "loss of appetite" OR "muscle pain" OR myalgia))) AND ((constipation OR nausea OR vomiting OR " abdominal pain" OR insomnia OR depression OR anxiety OR "decreased cognitive function"))) AND ((constipation OR nausea OR vomiting OR " abdominal pain" OR insomnia OR depression OR anxiety OR "decreased cognitive function")) Apply equivalent subjects on 2021-07-28 04:49 PM"	None	1500
Science Direct	29/07/2021	"long covid" OR chronic covid AND consequences	1 year	1160

PRISMA-P- supporting information

Click here to access/download
Supporting Information
PRISMA-P.docx
