# Supplementary material for: Characterisation of the long-term physical and mental health consequences of SARS-CoV-2 infection: A systematic review and meta-analysis protocol
Source: PLoS One. 2022 Apr 5;17(4):e0266232. doi: 10.1371/journal.pone.0266232 (PMC8982872; doi:10.1371/journal.pone.0266232)
Supplement: Attachment — Submitted filename: Response to Reviewers.docx [file pone.0266232.s004.docx]

| **Reviewer** | **Reviewer Comments** | **Responses to reviewer comments** |
| --- | --- | --- |
| #1 | Regarding the second outcome, Mental health outcome (Depression, anxiety) different studies may measure these outcomes differently or using different tools. How are you going to deal with this? | We plan to pool outcomes from different studies only if they were measured with similar tools. Where different tools are used, we will synthesize the results narratively.  (Line 204-205) |
|  | You may consider looking at the Grey literature as a means to reduce on the publication bias. | We have added Mednar database to source Grey literature. (Lines 136-137) |
|  | Its is good to specify the tools that you will use to assess the quality of the studies before hand (Of course different study designs require different tools) | The Newcastle-Ottawa Scale (NOS) quality assessment tool will be used to assess quality of cohort studies, and The Joanna Briggs Institute (JBI) Critical Appraisal Checklist will be used to assess quality of analytical cross-sectional studies.  (Line 194-196) |
| #2 | The authors should also consider searching for relevant papers through EMBASE and PsycINFO databases. | EMBASE and Psych INFO have been added to the list of databases to be searched. (Line 140-141) |
|  | Also specify whether you intend to apply any geographical or language restrictions | We will restrict the literature search to only studies published in English language with no restriction of geographical locations (Line 158-159) |
|  | What publication year/period do you intend to cover? e.g., 2020, 2021, etc? Mention it here | The literature search will be restricted to include studies published between March 2020 and March 2022.  (Line 131-133) |
|  | What does the author mean by social health domain? This seems out of place since this paper is about physical and mental health impact of Covid-19. | The social health domain has been removed  (Line 97) |
|  | search strategy: here, the authors listed the search terms they intend to use. How did they identify the Covid symptoms listed here? Was it through published key literature and/or through discussions with librarians, experts, etc? This should be stated. | The search strategy was developed after preliminary review of existing literature on the topic and in consultation with clinicians who routinely manage Long Covid patients. (Line 112-113) |
|  | But what if the patients have symptoms other than those listed in the search terms? It would be a good idea for the authors to seek external input/feedback on this e.g. from clinicians. | We are working in collaboration with clinicians who routinely manage Long Covid Patients |
|  | Appendix 2? No other appendix was mentioned prior to this one. The authors should crosscheck the numbering – edit or mention appendix 1 in the appropriate place. | Appendix 2 has been amended to appendix 1  (Line 131) |
|  | information sources: also consider searching EMBASE and PsycINFO, specify what publication years/periods you intend to cover and whether there will be geographical and language restrictions. | EMBASE and Psych INFO have been added to the list of databases to be searched |
|  | in line with the study title, the authors should clearly indicate when they are referring to physical vs mental symptoms. For example, “…… self-reported or verified physical symptoms of SARS-COV-2 infection……..”. | We are synthesizing data for self-reported symptoms and for verified symptoms. During analysis, we will separately synthesize self-reported and verified symptoms during. (Line 140-141) |
|  | host institution of the study may not be necessary. Consider extracting information about various definitions of post-acute covid, e.g., long covid or chronic covid. | Host institution of the study has been removed (Line 176). |
|  | study quality assessment: “The studies will be assessed for quality using quality assessment tools appropriate for each study design”. This statement is not sufficient as it does not tell readers which specific tool(s) you intend to use to undertake this task. Be specific. | Tools to be used for quality assessment have been listed accordingly. (Line 194-196) |
|  | descriptive analysis and meta-analysis: “data analysis” or “statistical analysis” would be a more appropriate name for this section. The authors should also consider conducting sensitivity analysis, if possible, to examine the effect of the definitions of post-acute covid e.g. >3 weeks (long covid) vs >12 weeks (chronic covid) vs both. This is important in view of lack of consensus regarding case definition which the authors mentioned in the introduction section. | “Descriptive analysis and meta-analysis” replaced with “data analysis” as suggested.  (Line 201).  Sensitivity analyses will be carried out to examine the effect of risk of bias on the pooled estimates and the timing of Long Covid symptoms reporting in order to refine the definition (Line 214-216) |
|  | Statistical analysis should also explore the presence/absence of pre-existing conditions. If there were pre-existing physical or mental health conditions, the persisting symptoms (during the post-acute phase) may not be exclusively due to the covid infection | Statistical analysis will be conducted to explore the presence/absence of pre-existing conditions in cases where data is reported. Studies that do not pre-existing conditions will be reported. (Line 219-219) |
| #3 | Authors to critically read through and make corrections to use of language. Authors could find useful other scientific research sites as contained in the attached document | We have formatted the manuscript according to PLOS one Journal specifications |
|  | In the space provided for the research question, please write it out in full. | Research question was written in full as suggested |
|  | Is the figure indicated (3000) expected funding? If so, please write the financial disclosure statement in full. | No specific funding was received for this study |
